# VarChat: the generative AI assistant for the interpretation of human genomic variations

**DOI:** 10.1093/bioinformatics/btae183

**Published:** 2024-04-05

**Authors:** Federica De Paoli, Silvia Berardelli, Ivan Limongelli, Ettore Rizzo, Susanna Zucca

**Affiliations:** enGenome srl, via Ferrata, 5, Pavia, 27100, Italy; enGenome srl, via Ferrata, 5, Pavia, 27100, Italy; Department of Electrical, Computer and Biomedical Engineering, University of Pavia, via Ferrata, 5, Pavia, 27100, Italy; enGenome srl, via Ferrata, 5, Pavia, 27100, Italy; enGenome srl, via Ferrata, 5, Pavia, 27100, Italy; enGenome srl, via Ferrata, 5, Pavia, 27100, Italy

## Abstract

**Motivation:**

In the modern era of genomic research, the scientific community is witnessing an explosive growth in the volume of published findings. While this abundance of data offers invaluable insights, it also places a pressing responsibility on genetic professionals and researchers to stay informed about the latest findings and their clinical significance. Genomic variant interpretation is currently facing a challenge in identifying the most up-to-date and relevant scientific papers, while also extracting meaningful information to accelerate the process from clinical assessment to reporting. Computer-aided literature search and summarization can play a pivotal role in this context. By synthesizing complex genomic findings into concise, interpretable summaries, this approach facilitates the translation of extensive genomic datasets into clinically relevant insights.

**Results:**

To bridge this gap, we present VarChat (varchat.engenome.com), an innovative tool based on generative AI, developed to find and summarize the fragmented scientific literature associated with genomic variants into brief yet informative texts. VarChat provides users with a concise description of specific genetic variants, detailing their impact on related proteins and possible effects on human health. In addition, VarChat offers direct links to related scientific trustable sources, and encourages deeper research.

**Availability and implementation:**

varchat.engenome.com.

## 1 Introduction

The rise of genomics and personalized medicine is generating a tremendous amount of data, with genomic variants as a primary research focus. These variants can be linked to disease susceptibility, drug responses, and other phenotypic outcomes (Karchin and Nussinov 2016), and the vast majority is well-documented in scientific papers.

Not just the identification of these variants from sequencing data, but also the effective curation and interpretation of this large amount of information may be challenging, and several methods have been proposed to automate this process (Nicora *et al.* 2022, Stenton *et al.* 2023, Zucca *et al.* 2024). Many efforts have been made to condense this knowledge in dedicated databases and publicly available resources, like ClinVar (Landrum *et al.* 2014), gnomAD (Karczewski *et al.* 2020), and OMIM (Hamosh *et al.* 2005).

The scientific literature, with its rich repository of knowledge, offers a wealth of insights into these genomic variants. However, the enormous volume of publications, coupled with the not yet perfect standardization in applying a nomenclature describing genomic variants, makes manual curation challenging (Lee *et al.* 2021). Furthermore, the enhancement of genomic variant discovery relies on the curation process, which is significantly improved by accessing not only the abstracts but also the full texts and supplementary data of scientific articles (Wei *et al.* 2013, Khare *et al.* 2014, Pasche *et al.* 2023).

Acknowledging this challenge, several tools have been developed to support genomic variant research in the scientific literature.

Among these, LitVar (Allot *et al.* 2018, 2023) is a semantic search engine designed specifically for linking genomic variant data in PubMed and PMC. By employing advanced text mining techniques, LitVar not only retrieves standardized variant information but also visualizes the relationships between variants and other associated entities, such as diseases and chemicals/drugs. Variomes (Pasche *et al.* 2022) is another tool designed as a high-recall search engine, focusing on aiding the curation of genomic variants. Different parameters allow for personalizing the search by specifying the timeline and adding keywords for papers re-ranking. Finally, SynVar (Mottaz *et al.* 2022) has been developed to ensure effective retrieval of variant-containing documents, providing descriptions in both standard and nonstandard formats found in the literature.

A significant limitation of these approaches is their inability to synthesize variants’ information into concise, human-readable texts that are suitable for clinical reports. Many systems prioritize data aggregation and categorization but fall short in generating comprehensive yet succinct textual interpretations.

Conversely, Large Language Models (LLM) based on generative AI, such as the widely recognized chatGPT (www.openai.com), Bard (google AI), Falcon (www.tii.ae), and Claude 2 (Anthropic, www.claude.ai), have the innate capability of comprehension and summarization of complex texts. These models are based on a deep learning architecture known as a Transformer (Vaswani *et al.* 2017), characterized by millions or billions of parameters, and from an innovative layer of “attention,” enabling the model to weigh the importance of different parts of the input differently, improving its understanding of context and relationships in the data. They have become integral to solutions widely used in our daily life and have demonstrated exceptional performance across multiple Natural Language Processing tasks, showcasing strong comprehension and reasoning abilities (Borji and Mohammadian 2023, Ye *et al.* 2023).

The balance between providing detailed and accurate genomic insights and ensuring readability for a diverse audience, including those without deep genomic and computational expertise, is a challenge yet to be fully addressed.

For this purpose, we introduce VarChat, the first generative AI based tool designed to search and summarize scientific literature about a human genomic variant and provide a concise text explaining the variant, insights from existing research, and associated references.

VarChat is freely available at varchat.engenome.com.

## 2 Materials and methods

VarChat requires as input genomic variants coordinates according to HGVS nomenclature (den Dunnen *et al.* 2016) together with gene symbols, or to dbSNP identifier. For every queried variant, VarChat produces concise and coherent summaries through an LLM model, enabling researchers and clinicians to capture the core insights of articles associated with these variants. In addition to textual summarization, the system provides the user with the 15 most relevant references, when available. The relevance of the publication is based on a modified version of the BM25 ranking algorithm, which primarily relies on the classic term frequency (Robertson and Zaragoza 2009). More weight is given to papers that cite the variant in the abstract and were published in the last two years, while papers that report the variant only in the supplementaries are penalized.

Furthermore, if the variant is present in ClinVar (Landrum *et al.* 2014), the corresponding records, the associated condition, the clinical significance, the review status and a direct link to the database page are provided. Finally, the translation of the answer in a different language (30 different languages supported) has been enabled.

VarChat graphical user interface is implemented in ReactJS and optimized for desktop and mobile, while the Restful API is built upon a serverless and scalable infrastructure leveraging on Amazon AWS Lambda functions, FastAPI and Python 3.

## 3 Results

**Figure 1.**
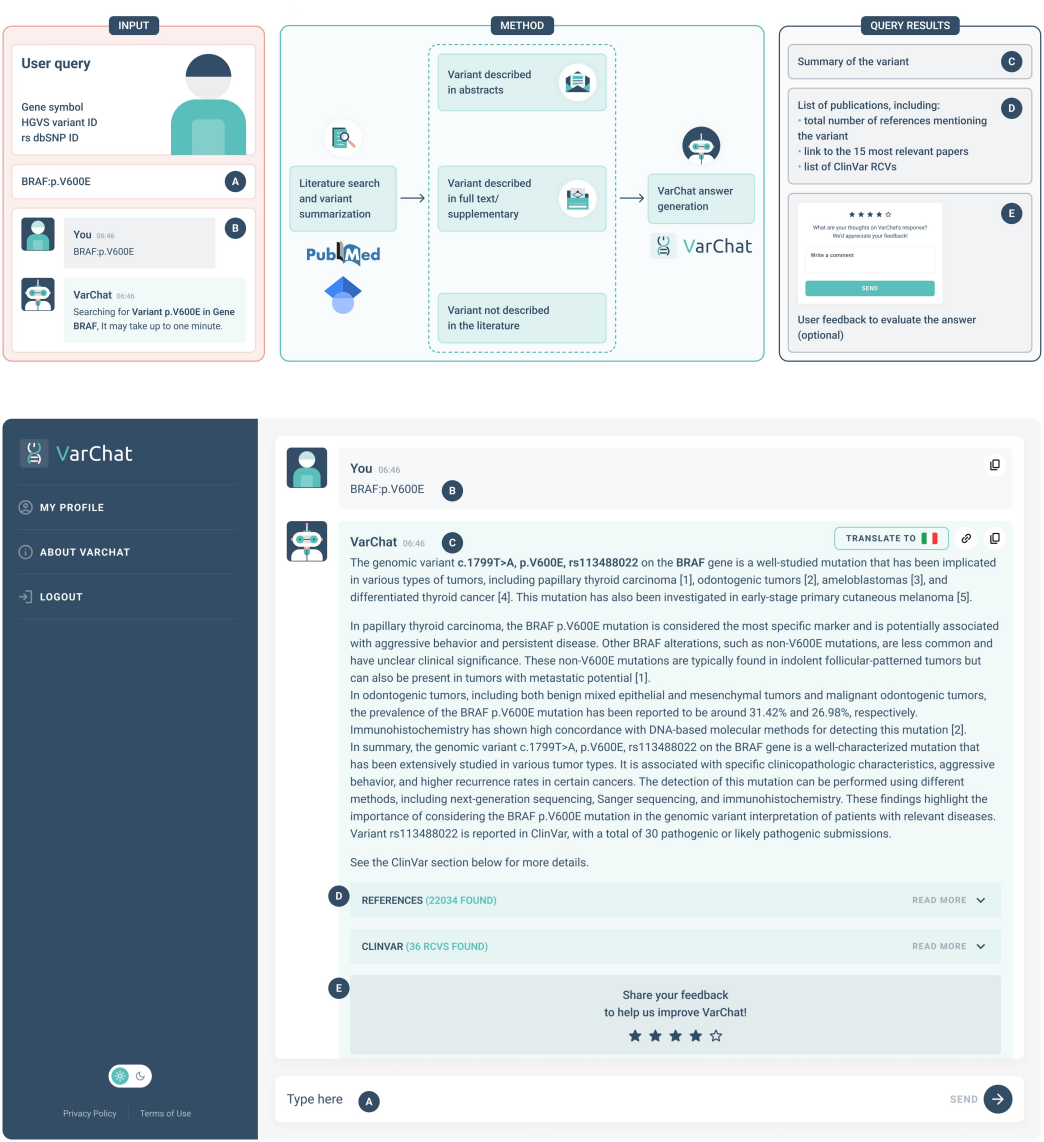
VarChat workflow and platform preview. (A) User prompt enabling variant’s query. Users can search genomic variants with HGVS nomenclature along with the gene symbol. (B) Variant searched by VarChat. (C) VarChat retrieves the literature associated with the searched variant and provides a comprehensive summarization. (D) If available, up to 15 references (sorted by relevance) are displayed, with direct links to PubMed. The total number of publications found is also shown. (E) Feedback system: users can evaluate the answer through a 5-star scoring system and provide feedback.

VarChat workflow is described in Fig. 1. Users can search genomic variants by HGVS nomenclature (den Dunnen *et al.* 2016), choosing between coding DNA reference sequence, protein reference sequence, mitochondrial DNA reference sequence or even both coding DNA reference sequence and protein reference sequence, together with the gene symbol. Examples of valid queries are: “BRAF:p.V600E,” “PINK1:c.926G>A,” “MT-ND4:m.11778G>A,” and “rs34637584.” For mitochondrial variants, we recommend using mitochondrial coordinates, such as “MT-ND4:m.11778G>A,” as an example.

Currently, genomic coordinates are not supported as input. HGVS or rs dbSNP id can be calculated for each variant through freely available annotation and interpretation software as VEP (McLaren *et al.* 2016) or conversion tools as TransVar (Zhou *et al.* 2015).

All the scientific papers mentioning the variant in the abstract, in the full text or in the supplementary information are retrieved. The 15 most relevant ones are shown to the user and the direct links to the full text papers are provided. The abstracts related to the searched variant are then exploited by VarChat for summarization purposes.

Based on the information at hand, VarChat generates a summary derived from the relevant publications and the insights of its LLM model. Specifically, if the variant is referenced in the abstract of PubMed or of freely available publications on Google Scholar, that text serves as an additional content for the summarization. If not, the response is entirely produced by the VarChat LLM. Regardless of the scenario, if there's a variant match with the scientific literature, the list of the corresponding references for the variant is displayed.

The system is designed to be trustworthy for users. Being an LLM-based system, VarChat can be prone to producing ‘hallucinations,’ a phenomenon where these models generate information that is not supported by the input data or is factually incorrect (Zhang *et al*. 2023). This aspect can be particularly challenging when LLMs are used for tasks that require high levels of accuracy and reliability.

To enhance the transparency of the process, VarChat clearly informs users about the source of its responses, indicating whether the answer was derived from references or generated solely from the knowledge of VarChat LLM.

After receiving a response, users have the option to provide feedback using a 5-star rating system and can also add a comment. This information will be exploited to fine-tune the system and identify key areas for improvement.

To the best of our knowledge, no similar tools currently exist.

### 3.1 Conclusions

VarChat is the first generative AI-based tool specifically designed to support genomic variant interpretation by efficiently finding and summarizing relevant scientific literature, thus acting as a genetic assistant.

VarChat holds the potential to serve the community of genetic professionals as a valuable aid in assessing human genetic variations through generative AI thus enhancing understanding of variants’ impact and their implications.
